# Lack of association between interleukin 28B gene polymorphisms (rs8099917G/T, rs12979860 C/T) and susceptibility to chronic hepatitis C virus infection, Tehran, Iran

**Published:** 2016-12

**Authors:** Maryam Karkhane, Seyed Reza Mohebbi, Pedram Azimzadeh, Mahsa Saeedi Niasar, Mohamad Reza Sarbazi, Afsaneh Sharifian, Afshin Mohammad Alizadeh

**Affiliations:** 1 *Basic and Molecular Epidemiology of Gastrointestinal Disorders Research Center, Research Institute for Gastroenterologyand Liver Diseases, Shahid Beheshti University of Medical Sciences, Tehran, Iran*; 2 *Gastroenterology and Liver Diseases Research Center, Research Institute for Gastroenterology and Liver Diseases, Shahid Beheshti University of Medical Sciences, Tehran, Iran*; 3 *Deputy of Public Health, Shahid Beheshti University of Medical Sciences, Tehran, Iran*; 4 *Bone Marrow Transplantation Department, Taleghani Hospital, Shahid Beheshti University of Medical Sciences, Tehran, Iran*

**Keywords:** Interleukin 28B, Hepatitis C, rs8099917, rs12979860, Hepatitis C susceptibility

## Abstract

**Aim:**

Chronic Hepatitis C infection is a critical health problem worldwide, which caused by hepatitis C virus (HCV). Interleukin 28B (IL28B) is a determinant factor in disease progression and also susceptibility to chronic HCV infection.

**Background:**

The most significant aim of this study is to analyze the association between IL28B gene polymorphisms with susceptibility to chronic HCV infection in Iranian population.

**Methods:**

This study follows a case- control study design, in which, 288 patients with chronic hepatitis C and 250 healthy individuals were genotyped for IL28B polymorphisms (rs12979860 C/T and rs8099917 G/T). Studied population collected from Taleghani Haospital, Tehran. Genotyping of IL28B gene polymorphisms were performed using PCR-Restriction Fragment Length Polymorphism (PCR-RFLP) method. 10 percent of the studied population was sequenced to validate the results.

**Results:**

rs8099917 G/T and rs12979860 C/T were differently distributed in hepatitis C patients and healthy controls in the female gender. TT, TG and GG genotypes distribution in the female gender were 56.7%, 39.8% and 4.5% in cases and 67%, 31.6% and 1.4% in controls (p=0.54). Also CC, CT and TT genotypes distribution were 31.8%, 61.4% and 6.8% in cases and 51.7%, 44.9% and 3.4% in controls (p=0.2). However, there was no significant difference in the allelic frequency and genotype distribution of rs12979860 C/T and rs8099917 T/G in both HCV patients with genotype 1a and 3a.

**Conclusion:**

It seems that rs8099917 G/T polymorphism plays a significant role in susceptibility to chronic HCV infection in Iranian population. On the other hand, no association was found between rs12979860 C/T polymorphisms and chronic hepatitis C.

## Introduction

Hepatitis C has become an alarming problem worldwide**. **170- 200 million people have been infected with hepatitis C virus (HCV) infection (1-4). HCV infection generallyleads to a chronic disease in most of the patients. These patients gradually face with hepatic inflammation and fibrosis and finally liver cirrhosis and/or hepatocellular carcinoma (HCC). HCV is also one of the main reasons of liver transplantation around the world (3, 5-8). Recent studies have found that viral, host, and environmental factors may involve in susceptibility to HCV chronic infection or spontaneous clearance of the infection (9- 11).

Among the host factors, single nucleotide polymorphisms (SNPs) near the IL28B gene, which encode the IFN-λ , is strongly associated with spontaneous clearance and sustained viral response (SVR) or non- viral response (NVR) to PEG- IFN-α and Ribavirin (1, 5, 11-17). Several SNPs were associated with treatment–induced and spontaneous clearance of chronic HCV, but the most recent studies accentuated on rs12979860 and rs8099917 SNPs where have been located into IL-28B in all of major ethnicities around the world (3, 12, 18, 19). However, the underlying biological mechanisms of this phenomenon are not well understood (20).

Although, T rs8099917 and C rs12979860, most strongly associated with HCV clearance, but it might be affected by the HCV genotype, racial diversity and population differences. The unfavorable IL28B polymorphisms are highly prevalent in African population in comparison to Asian and European which may correlate with higher susceptibility to hepatitis C infection and lower SVR rate in African- American patients under PEG/ IFN-α treatment (21). Herein, in the present paper, distribution of IL-28B rs12979860 and rs8099917 was compared with a healthy control group and patients with chronic HCV infection.

## Patients and methods

Study population

Cross-sectional and case-control study was done analyzing 288 adult patients with chronic HCV infection who admitted in Gastroenterology and Liver Diseases Research Center, Shahid Beheshti University of Medical Sciences, between 2012 -2014 and also 250 healthy individuals as a control group.

Control group were healthy adults without any liver diseases along with negative results for anti–HCV antibody and HCV viremia which tested by ELISA (DRG International Inc., USA) and Reverse transcription-PCR (RT-PCR) respectively. Selection criteria for patients group were positive results for anti–HCV antibody ELISA. RT- PCR and HCV RNA PCR were tested for anti HCV antibody positive cases. Co-infected patients with HBV and HDV were excluded from the study. These patients were cases with hepatitis B surface antigen (HBsAg) positive and/or anti- HDV antibody positive using by ELISA serological test (DRG International Inc., USA).

Qualitative and quantitative methods

**RNA extraction, RT-PCR and nested PCR**

Viral genomic RNA of HCV was extracted from 200µl of plasma with the QIAmp viral RNA minikit (Qiagen, Hilden, Germany). Complementary DNA (cDNA) was synthesized based on Romani *et al *with exception some modifications (22). This reaction was directed in a total volume of 20μL, consisting of 1 μL of random hexamer primers, 5 μL(100 ng) of ssRNA template, 4 μL of 5× buffer, 0.5 μL of Ribolock RNase inhibitor, 2 μL of dNTP mix, 1 μL (200 U) of RevertAidTM reverse transcriptase (Fermentas, Latvia). Reverse transcription was carried out at 42°C for 60 min. Nested PCR previously was described by Salehi Moghadam *et al *(23).

HCV-genotyping

HCV-RNA extraction and RT-PCR were followed by qualitative nested PCR based on Salehi Moghadam *et al *(23) and finally, genotyping of HCV was performed by direct sequencing (Macrogen Co, South Korea).

IL28B gene polymorphisms

Human genomic DNA was extracted from blood sample of both patients and control group by the standard phenol- chloroform method (24). Polymerase chain reaction (PCR) in PCR thermal cyclers (Eppendorf AG, Hamburg, Germany) was used to amplify a region of DNA surrounding of IL28B SNP by the specific designed primers (Table 1). PCR- restriction fragment length polymorphism (PCR-RFLP) was used for IL28B genotyping.

The resulting PCR products were digested with 10 units of endonuclease restriction enzymes for overnight and RFLP product separated onto 3% w/v agarose gel (Hoffmann la Roche AG, Basel, Switzerland) stained with ethidium bromide. NmuCI and BstUI (Fermentas, Vilnius, Lithuania) were used for genotyping of rs8099917 T/G and rs12979860 C/T respectively. Validation of resultant genotypes was performed by sequencing ten percent of sample via ABI genetic analyzer 3130xl.

Statistical analysis

Hardy- Weinberg equilibrium was used for evaluation of genotype distribution. Results were analyzed by comparing allelic frequencies (ratio of the test allele to total alleles), genotypes and the carriage rates (number of individuals with at least one copy of the test allele) in different populations. Differences were analyzed by Chi-square and t tests. Odds ratios (OR) and the confidence intervals (95% CI) of the ORs were calculated by logistic regression analysis. All of statistical analysis were two sided, and p<0.05 was considered significant.

## Results

The mean age of the patients was 45.45±0.56 and in the healthy control group was 43.54±0.65 (P-value=0.02). Male was the dominant gender in the patient group, in versus of the control group which female gender was dominant. The patient’s profiles are presented in Table 2. However, the confounding effects of age and gender were omitted by logistic regression analyses.

Genotype distribution of rs12979860 C/T and rs8099917 T/G were consistent with Hardy-Weinberg equilibrium in healthy controls (P=0.17 and P=0.32). We compared the distribution of IL28B genotypes between the healthy group and the HCV infected patients. The frequency of IL28B rs12979860 CC, CT, and TT genotypes in chronic hepatitis C patients was 46.9%, 44.8%, and 8.3% and in healthy individuals was 47.6%, 46.8%, and 5.6%. In addition, the frequency of IL28B rs8099917 TT, GT, and GG genotypes in chronic hepatitis C patients was 61.6%, 34.5%, and 3.8% and in the healthy individuals was 62.6%, 35% and 2.4%. Allelic frequency and genotype distribution were illustrated in Table 3.

**Table 1:** Sequence of Primers used in PCR

**Gene polymorphisms**	**Primer Sequence**	**PCR Ann**. **temp.**	**Amplified Fragment size**
**IL28Brs8099917**	5-AGTAAGTCTTGTATTTCACCTCC-35-TATCCTAAATTGACGGGCCATC-3	63°c	237bp
**IL28Brs12979860**	5-GCTTATCGCATACGGCTAGG-35- AGGCTCAGGGTCAATCACAG-3	60°c	242bp

**Table 2:** Characteristics of studied population

**Criteria**	**Patients**	**Healthy controls**
**Age, mean ± SD, year** **Sex (male/female)%**	45.45 ± 0.56 84.70 - 15.30	43.54 ± 0.65 41.20 - 58.80
**Age range, year**	15 - 89	16 - 82

**Table 3:** IL28B rs12979860 and rs8099917 genotype and allelic frequency

**Variable**	**Cases (n=288), No (%)**	**Controls (n=250), No (%)**	**Adjusted ** **a ** **OR (95% CI), P value**
**rs12979860 C/T**			
**Genotypes**			
**CC**	46.90	47.60	Reference (the category which presumed had the lowest susceptibility)
**CT**	44.80	46.80	1.03(0.78-1.36), 0.82
**TT**	8.30	5.60	1.13(0.65-1.96), 0.64
**Alleles**			
**C**	69.30	71.00	Reference
**T**	30.70	29.00	1.04(0.77-1.40), 0.76
**rs8099917 G/T**			
**Genotypes**			
**TT**	61.60	62.60	Reference
**GT**	34.50	35.00	0.95(0.74-1.26),0.72
**GG**	3.80	2.40	1.23(0.55-2.76),0.60
**Alleles**			
**T**	79.00	80.00	Reference
**G**	21.00	20.00	0.98(0.70-1.37), 0.93

**Table 4:** Genotype distribution and allele frequency of IL28B polymorphisms based on gender

**Variable**	Male			Female		
	**Cases, No(%)**	**Controls, No, (%)**	**Adjusted ** **a ** **OR(95%CI), P-value**	**Cases, No,(%)**	**Controls, No, (%)**	**Adjusted ** **a ** **OR(95%CI), P-value**
			**rs12979860 C/T**			
**Genotypes, No,%**						
**CC**	49.60	41.70	Reference	31.80	51.70	Reference
**CT**	41.80	49.50	0.70 (0.5-0.99), 0.04	61.40	44.90	2.36(1.40-3.97), 0.001
**TT**	8.60	8.70	0.78(0.42-1.44), 0.44	6.80	3.40	3.29(1.09-9.88),0.03
**Alleles, No, %**						
**C**	70.50	66.50	Reference	62.50	74.10	Reference
**T**	29.50	33.50	0.81(0.57-1.16), 0.26	37.50	25.90	1.77(1.06-2.94), 0.02
			**rs8099917 T/G**			
**Genotypes, No,%**						
**TT**	62.70	56.30	Reference	56.70	67.00	Reference
**TG**	33.60	39.80	1.3(0.93-1.84), 0.11	39.80	31.60	1.54(0.93-2.54),0.09
**GG**	3.70	3.90	1.02(0.42-2.64), 0.96	4.50	1.40	4.18(1.00-17.46),0.04
**Alleles, No,%**						
**T**	79.50	76.20	Reference	76.1	82.70	Reference
**G**	20.50	23.80	0.814(0.55-1.20),0.30	23.9	17.30	1.51(0.85-2.70),0.15

**Table 5:** Genotype distribution and allele frequency of IL28B polymorphisms based on HCV genotypes

**HCV genotype**	1a	3a	other	Unadjusted P-value
		**rs12979860C/T**		
**Genotypes,%**				
**CC**	38.80	50.70	43.90	
**CT**	51.30	38.80	46.30	0.29
**TT** **Alleles,** ** No,%**	10.00	10.40	9.80	
**C**	103(64.4)	94(70.1)	55(67.1)	
**T**	57(35.6)	40(29.9)	27(32.90)	
		**rs8099917T/G**		
**Genotypes,%**				
**TT**	50.00	60.40	68.30	
**TG**	43.80	35.10	26.80	0.08
**GG** **Alleles,** ** No,%**	6.30	4.50	4.90	
**T**	115(71.90)	105(78.40)	67(81.70)	
**G**	45(28.10)	29(21.60)	15(18.30)	

The results showed that differences in the distribution of IL28B rs12979860 C/T genotype between patients with chronic hepatitis C and healthy individuals was not significant (P=0.20), and also the rs8099917 wasn’t distributed differently in population (P=0.54).

**Figure 1:**
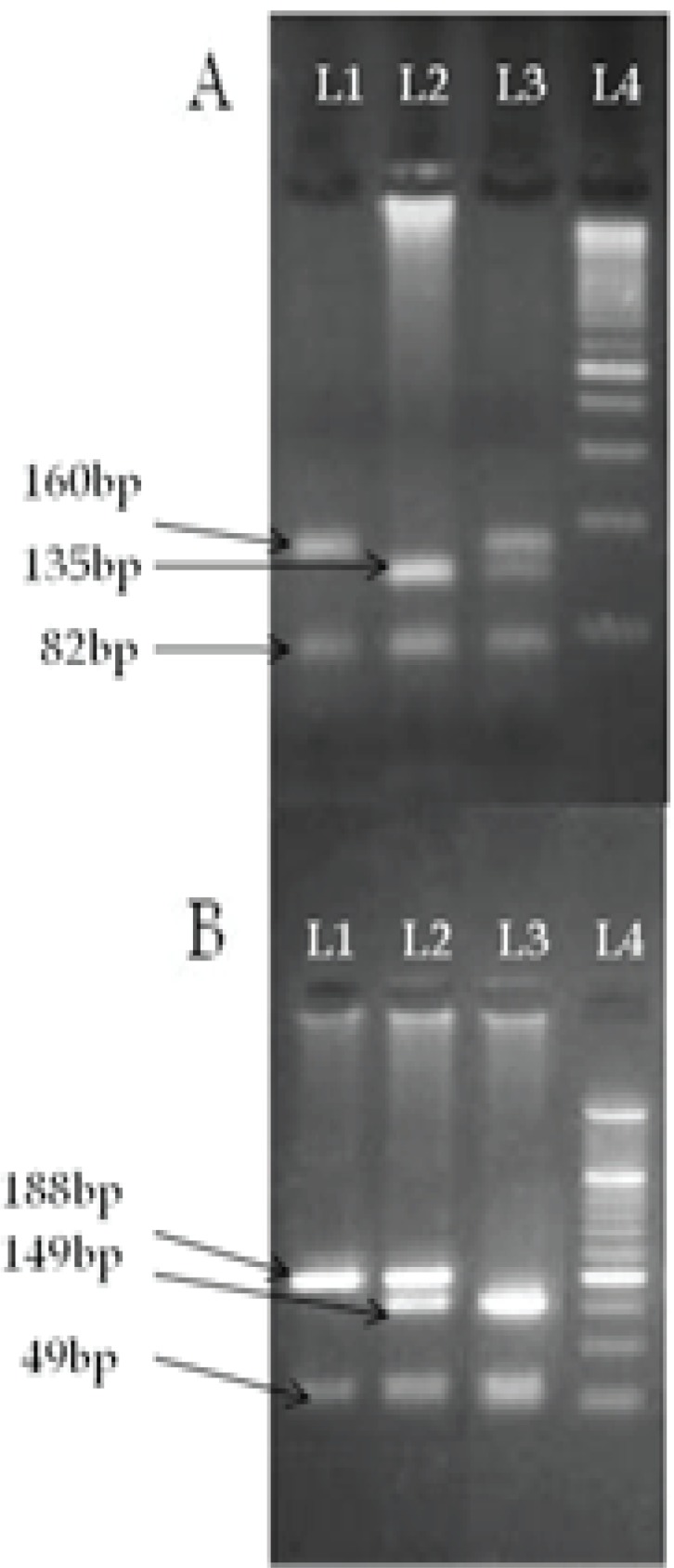
A: rs12979860 fragments. L1: TT genotype. L2: CC genotype. L3: CT genotype. L4: 100bp DNA ladder. B: rs8099917 fragments. L1: TT genotype. L2: TG genotype. L3: GG genotype. L4: 50bp DNA ladder

The rs12979860 C and rs8099917 T allelic frequencies were increased in thehealthy control group versus rs12979860 T and rs8099917 G alleles respectively. In present study, the distribution of alleles of rs12979860C/T and rs8099917T/G was in accordance to Hardy-Weinberg equilibrium in patients group (P=0.59 and P=0.70) and nearly similar distribution was observed in healthy controls (Table 3). Table 4 shows data illustration in both genders. More analyses in male and female separately showed unlike male gender, IL28B rs8099917 T/G and also IL28B rs12979860 C/T genotype had significantly different distribution in case than a control group of the female gender (P=0.05 and P<0.01). It is also noteworthy that a number of individuals with at least one copy of the rs12979860 C allele was higher than healthy people in female gender (P=0.02). Allele distribution of IL28B rs12979860 and ra8099917 was illustrated in table 5. However, there wasn’t any significantly different distribution between patients with various HCV genotypes. The pattern of electrophoresis of resultant PCR and RFLP products are depicted in figure 1, whereas resultant RFLP genotyping was assayed and validated by direct sequencing of PCR products.

## Discussion

Since 2009, several studies have determined a strong association between IL-28B polymorphisms with spontaneous and treatment induced clearance of HCV (3, 12-14, 16, 19, 21, 25-29). IL28B SNPs as the strongest predictor of spontaneous and SVR-induced treatment, differently distributed in chronic HCV infected patients and healthy controls (29-32). For these reasons, distribution of IL28B SNPs in population and its confounding role in HCV susceptibility were challenged in this study.

IL28B polymorphisms were observed with different distribution between racial groups (33). Rs8099917 TT and rs12979860 CC polymorphisms are the most frequently observed in Asian population and the lowest in African- American which can be related to the different SVR rate in various populations (30, 34-37). In the present research which performed on an Iranian population, different distribution of IL28B polymorphisms was observed in males and females. Multiple studies were demonstrated the association of rs12979860 CC genotype with spontaneous clearance of HCV in Asian population (29, 38). On the other hand, several studies showed that rs8099917 TT genotype was common in Asian population and effects on spontaneous clearance of HCV (13, 17, 32, 39).

Treatment response of the HCV patients was also influenced by IL28B genotype. In this regard, several studies showed the correlation between IL28B polymorphisms and treatment response in HCV patients (15, 17, 40). Recently Coppala et al (2013) was observed that rs12979860 CC genotype effects on treatment related clearance of HCV in HCV-HBV co-infected patients (41).

The present research demonstrated the correlation between IL28Brs8099917 T/G and IL28Brs12979860 C/T polymorphism and hepatitis C susceptibility in an Iranian female population. However, any association wasn’t found between IL28Brs8099917 T/G and IL28Brs12979860 C/T and HCV susceptibility at male gender, and it was seemed that rs12979860 T allele can significantly influence to HCV susceptibility at female gender in this work.

In accordance with observation, the prevalence of rs8099917 GG and TG genotype in female HCV patients was increased in comparison with the control group and G allele was higher in patients group than healthy controls which are explain patients’ susceptibility to HCV infection in female gender. This observation is consistent with recent GAWAS about association between rs8099917 T/G and HCV spontaneous clearance in different ethnicities (29). In addition, higher frequencies of IL28B rs12979860 CT and TT were observed in HCV patients just in female gender. The results of the study was consistent with previous studies were reported IL28B rs12979860 C and rs8099917 T protective alleles to be more frequent in East Asia (42). The rs8099917 T and rs12979860 C alleles frequency were 79 and 69.3 in Iranian patients with hepatitis C. These results were resemble with Sharafi et al with 77.4 rs8099917 T and 63.9 rs12979860 C in Iranian hepatitis C patients, and also with 63.4 rs12979860 T allele frequency in European–American population by Ge et al. (40, 43). Allele distribution in genotype 1a and 3a were similar to Sharafi et al (44). It is seemed that rs12979860  C and rs8099917 T protective alleles in genotype 3a were higher than genotype 1a.

The association between genetic variants, inadequate serum cytokines, inappropriate immune response and susceptibility to various diseases is a topic assay which has been investigated intensively for hepatitis C. Scientists and researchers now believe that IL28B polymorphisms are the strongest predictors of sustained virological response to HCV. In addition, IL28B polymorphisms can be affected on susceptibility for HCV infection in different populations. IL28B polymorphisms may be associated with mRNA expression and correlated with host protection against HCV infection. IL28B rs8099917 and rs12979860 is distributed differently in female patients with chronic HCV and healthy controls in Iranian population. Rs12979860 T allele may increase the susceptibility to hepatitis C infection and decreasing possible spontaneous clearance just in the female gender. However, this survey was influenced by viral load and HCV genotype. Therefore, IL28B can be assumed as a determinant factor in hepatitis C susceptibility in Iranian population especially in the female gender.
